# Pathology and Treatment of Milk-Sickness

**Published:** 1867-01

**Authors:** A. D. Cosby

**Affiliations:** Calhoun, Ky.


ATLANTA

### Medical and Surgical Journal.

NEW SERIES.

Vol. VIII. JANUARY, 1867. No. 11.

### ORIGINAL COMMUNICATIONS.

### ARTICLE I.

*Pathology and Treatment of Milk-Sickness.* By A. D. Cosby, M. D., of Calhoun, Ky.

A few days since I met with the Atlanta Medical and Surgical Journal, in which I found an able article, written by Dr. John M. Johnson, of that city, on the Pathology and Treatment of Milk-sickness. I read the article with much pleasure, not only on account of the interesting matter it contained, but also, on account of its author, who is an old friend of mine, whom I first met in 1840. In that year I located near the town of Rumsey, on Green River, where he then resided, for the purpose of practicing my profession. I have continued to practice in this locality ever since, and, as it is near the centre of the milk-sick region, of which he speaks in his article, lying between Green River and Panther Creek, I have had an ample field in which to make observations, and flatter myself that I have learned some very important facts in regard to the disease and its treatment. The result of my observations I present to the readers of the Atlanta Medical and Surgical Journal, not only for the benefit of my medical brethren whose experience has been confined to regions in which the disease never prevailed, but also for the purpose of confirming the correctness of the pathological views expressed by my friend Dr. J., who manifests on this, as on all subjects connected with the profession, a becoming zeal.

I do not propose to speak of the origin or cause of milk-sickness. Of this I know nothing more than was known twenty-five or thirty years ago, and consequently could not throw new light upon that subject. I am inclined to think, however, that it grows out of some cause peculiar to the native forest; as cattle that are confined in their grazing, to cleared and cultivated soil, are never known to have the *trembles*. The Doctor's mushroom theory is embraced by many, and stands the test of criticism as well, if not better than any of the various theories advocated by the physicians and people in this region of country.

It is evident that milk-sickness is produced by a non-volatile poison, which enters the system through the stomach, and from thence is conveyed to, and expends itself upon the nervous centres. Of the nature of this poison, nothing is known, except what has been learned from the symptoms of the disease which it produces.

The first and most prominent symptom manifesting the existence of the poison in the system, is extreme debility, amounting, in the muscular tissues, almost to paralysis. Hence it is, the muscular system of a person, affected with the poison, becomes fatigued and exausted on the slightest exertion. A walk of fifty yards will so completely exhaust the sufferer that he will be compelled to sit, or lie down, to rest for a few moments, before he can proceed, feeling as well otherwise as usual. This condition of the system is denominated the *tires*, which may be considered the first stage of the disease. In some instances it lasts but a few hours, while in others it endures for eight or ten days, when it either passes off without any further evil consequences, or merges itself into a fully developed case of milk-sickness. The length of time this symptom may continue will depend upon the balance maintained between the accumulation of the poison and the tolerance of the system for it,

When accumulation exceeds the tolerance of the nervous system for the poison, the mutiny sets in, and the conflict begins. This brings on the second stage, every symptom of which indicates, beyond all question, a diminution of the vital forces far below the normal standard.

Nausea and vomiting are the first and most annoying symptoms of the second stage, which continues until the patient dies, or the disease is broken up.

As Dr. J. informs us in his article, the vomiting is not the result of vascularity from phlegmasia of the sanguiferous system, but from a deranged condition of the nervous centres.

But whether the vomiting is the result of a paralyzed condition of the stomach, which only acts when it is full and can contain no more, as Dr. J. asserts, or whether the nerves of the stomach are rendered irritable from debility, resulting from the pernicious effects of the poison, is a question of but little importance, when considered with reference to the treatment, as the remedies that would relieve the one condition of the stomach, would remove the other. Certain it is, however, that the secretions of the stomach are very copious and irritating in this disease, either from increased and morbid action of the stomach, or for the reason that they are all retained, by not being permitted to pass into the intestines through the pyloric orifice of the stomach. Besides all the bile which may be secreted, instead of passing away through the bowels, is regurgitated into the stomach, and adds much to its irritating contents.

Obstinate constipation is another symptom of the second stage. It always occurs after the vomiting sets in, and never before. These two symptoms seem to synchronize.

The reasons for this are obvious. The vomiting prevents the gastric juice, the other contents of the stomach, and all the bile secreted, from passing into the alimentary canal, upon the stimulating effects of which the bowels depend for their healthy action.

Again, it is in the intestines that the poison of milk-sickness is separated from the organized matter through which it is introduced into the system, and it no doubt produces its poisonous and pernicious effects, to a greater or less extent, on the nervous and muscular coats of the intestines, before it gets into the blood, and reaches the nervous centres, through which it acts on the system in general, and thus establishes a local, as well as a general effect on the bowels. It will also be remembered that in all diseases in which vomiting occurs as the result of disturbance upon the brain, that obstinate constipation always exists.

There are many other symptoms of the first and second stages, such as weariness and lassitude, fullness of head, thirst, pulse a little full, but not accelerated, diminished warmth of the body and extremities, unpleasant and peculiar odor of breath, a feeling of despair, dry skin, tongue coated with a dark slime, or unusually clean; the latter symptom denoting a stubborn case, as it is always accompanied with a quick pulse, violent vomiting and throbbing of the aorta, which indicate more inflammation about the stomach, or duodenum, than is usual in the disease. But the vomiting and constipation are the symptoms to which we have to direct our remedies, and upon the relief of which the other symptoms all disappear, except in cases where fatal lesions have occurred.

I adopted, in 1849, the pathological views of this disease, so clearly expressed by Dr. Johnson, and which have been merely recapitulated by myself, and resolved to try a treatment predicated upon them.

It readily occurred to me that the first indication was to counteract the debilitating effects of the poison on the nervous system: secondly, to remove the poison from the system, through the emunctories.

Sulphate of quinine was the remedy which I selected as most likely to fill my first indication, and it was not long before I had an opportunity to try its effect. I was called to see Mrs. Chapman, (wife of John Y.,) who lived in Daviess county, in consultation with Dr. De Moss. Upon my arrival, I learned that she had been laboring under the disease of milk-sickness for nine days, during which time the Doctor had been treating her case without affording the slightest relief. She had all the symptoms of the disease, unmitigated in their force. The vomiting was obstinate. Not the slightest peristaltic movement had taken place in the bowels. The pulse was weak and slow; body and extremities cool; and every symptom indicated increasing debility.

Without addressing the Doctor any reason for the proposition, I proposed to give sulphate of quinine, to which he readily consented, by saying that he would not oppose any remedy I might offer, as he had exhausted his skill, and sent for me because he felt unable to do anything more in the case, but asked me how I expected to get the stomach to retain the quinine long enough for it to have its specific effect on the system. I told him that I thought we could suspend the vomiting for three or four hours by putting the third of a grain of morphine on the surface of a blister which he had drawn over the epigastrium, as it always had that effect. The stomach soon became quiet, and we gave her three 10 grain doses of quinine, two hours apart. Her extremities and general surface became warm, and to my great joy she never vomited another time during her sickness.

We gave a purgative preparation, composed of spirits of turpentine, castor oil, and croton oil, which moved the bowels in the usual length of time for such a purgative. A repetition of a mild purgative from time to time, as circumstances seemed to demand, completed the cure. A sore mouth, produced by the mercury which the Doctor had given her before I was called in, constituted the most annoying symptom with which we had to contend during her convalescence.

Now, it is evident that the vomiting, in Mrs. Chapman's case, was not produced by inflammation, because we know that quinine will not cure inflammation of the stomach in so short a time, or in fact not at all. The truth is that in that case the morphine given rendered the nerves of the stomach insensible to, and the quinine, when it produced its specific effect, raised them superior to the irritating contents of the stomach, and thus gave relief by restoring to the entire nervous system its usual tone; or, in other words, by overcoming the debilitating effects of the poison.

I hope that I may be indulged while I give a brief history of the next case, in which I administered quinine, as it illustrates, very strikingly, not only the completeness of the relief which it gave, but also the calming and soothing influence it exerted over the nervous system. I was called on to visit Mrs. Mitchell, at the onset of her disease, and as I did not wish to experiment unnecessarily in her case, I treated her on my old plan, for five days, but without affording any relief. I resolved to resort to the use of quinine again, and adopted the same plan pursued by me in Mrs. Chapman's case, which resulted in giving similar relief. The relief was so sudden and unexpected to her female friends and relatives who were in attendance, that it excited their apprehensions to such a degree, that they supposed the patient, using their own language, "to be struck with death." Her husband, partaking of the same apprehensions, came to me with a proposition to call in consultation. To this I objected, giving as my reason, that his wife was better; that the effects which he witnessed were the result of the remedies which I had administered, and that I entertained not a doubt of her recovery. These statements quieted him for a short time; but the ladies and his wife's calmness soon aroused his fears again, and he renewed his proposition for consultation. I asked him to name the physician for whom he proposed to send. He said Dr. L., of Sacramento. To this I replied, that I had no objection, but as he was alarmed about his wife's case, I hoped that he would allow me to send to Owensboro' for Dr. S.

The messengers were dispatched, and the consulting physicians arrived in about seven hours. They examined the case together, and decided that her calm and quiet condition was the result of opiates. I assured them that I had not given her any opium internally, but had sprinkled the third of a grain of morphine on the surface of a blister which had been drawn over the stomach, the effects of which I supposed had passed away, as twelve or thirteen hours had elapsed since its application. The purgative which I had given her before the consulting physicians were sent for, operated, and her convalescence was rapid.

I did not inform my consulting brethren that I had given my patient thirty grains of quinine; first, because I regarded my patient's recovery as certain, and was satisfied she would lose nothing by my silence; second y, because the efficacy of quinine as a remedy in milk-sickness was a discovery of my own, and I did not wish it to pass into the hands of others before I had ample time to establish both its efficacy and its safety; thirdly, because I knew that if she were to die from any cause, however remote from the disease for which I was treating her, less obvious in its nature and effects than a bolt of thunder, that her death would be attributed to the remedy, and that I would fall a victim, all bleeding and torn, at the feet of a set of merciless persecutors.

This language may sound strange to the physicians of this day, and of other sections of the country; but it is certainly just in its application to a large number of the profession who figured conspicuously during the prevalence of milk-sickness in this locality. The highest ambition of he profession was to aspire to the attitude of a Magnus Appollo, or the leadership of the treatment of this disease. This was very natural, and, I must add, justifiable, as such a position opened up an extensive and lucrative field of practice in this disease alone; and then, it was reasonable for the public to conclude, that if a physician possessed skill and sense enough to cure milk-sickness, he would do to depend upon to treat successfully any other disease to which human nature was heir. Hence the spirit of rivalry raged high, and often led members of the profession to do and say things which savored more of selfishness than of justice and equity.

But thank the Lord! the conflict is over. The calm days of peace have dawned upon our suffering profession once more. The treatment of the disease is understood, and its occurrence becomes less and less frequent with each successive year, and as the arts of civilization make their inroads upon the native forest. It can now be declared, as a truth, that milk-sickness produces but little more excitement in the profession than does intermittent fever or the Lincoln itch.

I hope that I will be pardoned for this digression. There are a few other points of some importance which I desire to notice. Whisky will relieve the vomiting in many cases, by its stimulating effects on the nervous system. It is most reliable in those cases in which the poison remains in the system but a short time before the tolerance of the nerves gives way, and the disease is fully developed. I was sent for by Mr. Robert Hunt, in haste, and upon my arrival found three of his family vomiting from milk-sickness. They were all taken about the same time on that day. I gave them prepared chalk and whisky toddy freely. The vomiting ceased in about three hours. A purgative was given, which acted on the bowels, and relief followed in twenty-four hours.

Now, that these three cases were cases of milk-sickness, and that the poison had been in the system but a short time, is evident from the following facts. Just thirty-six hours before Mr. Hunt's family were taken sick, he drove up a herd of cattle which had been running at large in the woods during the summer. Among the number were a cow and calf which were separated; the calf turned into a dry lot, and the cow in a fresh stalk field. The milk of the cow was used by the family. A few days after the recovery of my three patients, the calf was seized with the trembles and died.

I have already observed, that the first stage of this disease lasts in some instances for eight or ten days before the second stage sets in. Tonics and stimulants will always cure the first stage, and thus cut short the disease. They should precede a purgative, or be given in connection with it, as purgatives given alone in the first will often bring on the second stage in all of its violence.

Cook's Pills, or any other purgative, taken at bed time, will, when they act the following morning, frequently stop the vomiting, and check the peristaltic action of the bowels. This is done by the debilitating effects of the purgative, which enables the poison to triumph over the tolerance of the system for it.

That tonics and stimulants constitute the proper remedies for the treatment of milk-sickness is further evident from the superior efficacy of the Miller over the Trafton treatment, as given by Dr. Johnson. The Trafton treatment consisted of the free use of effervescing drinks, etc., which possessed no stimulating power. The Miller prescription being composed of

Venice Turpentine ℥ ss.
Compound Spirits Lavender ℥ i.
Castor Oil ℥ ii.

contains two stimulating articles which enable the stomach to retain the remedies until they have time to pass into the alimentary canal, when they stimulate and move its peristaltic action, and thus disgorge the stomach of its irritating contents.

The compound spirits of lavender, which, it is said, was thrown in as a stimulating aromatic only, gives efficacy and value to the prescription.

Dr. Johnson informs us that he commenced the treatment of milk-sickness with a stimulant emetic of enpitorium and penniroyal or mint tea. Such an emetic doubtless does good, but let me suggest that a non-stimulant emetic should never be given in this disease. They invariably do harm, by increasing the prostration of the system. This reminds me of a few items of my early history, in connection with milk-sickness and its treatment, which I desire to give, first, to illustrate my former ignorance of the disease, and secondly, to show the difference between the effects of a stimulant and non-stimulant emetic in its treatment. In 1841, I

was sent for to see my first patient with milk-sickness, which was in the person of Mrs. Buck Gibson, who was laboring under a fourth attack of that disease. Dr. Eldred Glover had treated her in her former troubles, but was then dead. Mrs. G. had but little confidence in any other physician, and of course expected to die. She sent for me, I suppose, because I lived near by, and because my father, who lived in an adjoining county enjoyed a considerable reputation in the treatment of milk-sickness. I prescribed two grains of ipecac to be given in about four tablespoonsful of tea of capsicum. This was repeated every two hours for twelve hours, when a full dose of ipecac was given, with the free use of the tea. After the action of the ipecac her stomach became quiet, during which time it retained a sufficient quantity of purgative medicine to fully open the bowels. Aunt Mima (an abbreviation of Mrs. G.'s Christian name) recovered in a few days, and I have enjoyed her confidence ever since. But my folly consisted in attributing the beneficial effects of the capsicum to the ipecac. Hence it was, when I was called to see my next case, having no capsicum with me, I gave the ipecac alone, which evidently injured my patient materially, whereupon I abandoned the treatment.

But I must notice one effect which quinine invariably produces when given in milk-sickness, in doses sufficiently large to stop the vomiting. The patient always faints and falls over when occupying the erect position. Hence it is not safe to allow him to get out of bed to stool. He should use the bed-pan. The only patient I have lost since 1849, from this disease, died from this cause. I visited Mr. W. M., and prescribed five-grain doses of quinine, in combination with two grains of camphor, to be repeated every three hours until five doses were taken. On my return I found his stomach quiet, and the general surface and extremities warm. Prescribed castor oil and turpentine as a purgative, with directions not to get out of bed when it acted on the bowels, as he would surely faint, which might endanger his life. But he disregarded my orders, and against the remonstrances of his wife, got out of bed, fainted and fell prostrate on the floor. Heavy congestion ensued, from stagnation, and he died in a few hours.

The reason of this fainting is obvious. Quinine, when given in full doses, diminishes the propelling force of the heart, and thus adds to the debility produced by the poison, which renders the heart unable to supply the brain with blood against gravitation when the patient occupies an erect position. It is true that quinine warms up the extremities and the general surface; yet it does not accomplish this by increasing the power of the heart's action, but simply by its retaining effects: by overcoming the spasmodic stricture of the capillaries, and thus removing all obstructions to the free circulation of the blood on the general surface.

After the use of quinine, the patient should be confined to a horizontal position, and during the administration and action of purgatives, whisky toddy should be freely given, when no apprehension of fainting need be entertained. Mild chloride of mercury will, in many cases, facilitate convalescence by its action on the liver, but should never be given while the vomiting and constipation continue, as it is liable to produce ptyœlism, which always does harm, by increasing the debility and the irritating contents of the stomach. Stimulants and tonics supersede the necessity for mercury in a large majority of cases.

